# A Case of Anomalous Systemic Arterial Supply to the Posterior Basal Segment of Normal Lung in a Patient with Dextrocardia

**DOI:** 10.70352/scrj.cr.25-0062

**Published:** 2025-07-09

**Authors:** Aya Yamamoto, Kantaro Hara, Hidetoshi Inoue, Michihito Toda, Ryuichi Ito, Takuya Tanimura, Ryuhei Morita, Shoji Hanada, Takashi Iwata

**Affiliations:** Department of General Thoracic Surgery, Kansai Rosai Hospital, Japan Organization of Occupational Health and Safety, Amagasaki, Hyogo, Japan

**Keywords:** dextrocardia, anomalous systemic arterial supply, surgery

## Abstract

**INTRODUCTION:**

Dextrocardia, a condition characterized by the heart’s anomalous positioning to the right, is frequently associated with venous abnormalities, but arterial anomalies are rare. In particular, systemic arterial supply to the posterior basal segment of a normal lung in dextrocardia is an exceedingly rare finding, with only one previously reported case.

**CASE PRESENTATION:**

A 72-year-old female with a history of colorectal and gastric cancer surgeries presented with an abnormal chest X-ray showing a left lung shadow. She was asymptomatic, with no hemoptysis, cough, or dyspnea. Contrast-enhanced chest CT revealed dextrocardia and a thick anomalous artery branching from the aorta, running as the 9th intercostal artery, and supplying the S10 region of the left lung. Venous drainage was through a hypertrophic V10 branch of the pulmonary vein, with no evidence of sequestration or arteriovenous fistulas. Bronchoscopy excluded bronchial anomalies, confirming the diagnosis of anomalous systemic arterial supply to the posterior basal segment of a normal lung. Due to the patient’s carotid arteriosclerosis and risk of fatal hemoptysis, video-assisted thoracoscopic surgery was performed. The anomalous artery was transected, and visibly engorged pleural regions were partially resected. The procedure was completed in 46 min with minimal blood loss. Histopathology showed normal lung tissue with vascular wall thickening. Follow-up imaging after 2 years revealed no vascular abnormalities, and the patient remains healthy 7 years postoperatively, with no aneurysmal changes at the surgical site.

**CONCLUSIONS:**

We experienced a pulmonary artery originating from the aorta with perfusion only in a part of the basal segment of the lung complicated by a right thoracic heart. When preoperative angiography showed only segmental stain, it is considered safe and sufficient to resect only to the extent of the surface vasodilatation grossly during surgery.

## INTRODUCTION

Dextrocardia, anomalous positioning of the heart deviating to the right, is often associated with abnormalities of the great venous system, such as bilateral superior vena cava, azygos continuation due to inferior vena cava agenesis, and an enlarged coronary sinus. However, abnormalities in the arterial system are rare. According to Nair et al.,^1)^ cases of pulmonary artery anomalies associated with dextrocardia are uncommon. To the best of our knowledge, only one other case of a pulmonary basal segmental artery originating from the aorta in right-sided dextrocardia has been reported.^2)^ Moreover, cases where the anomalous artery perfuses only 1 segment of the lung, rather than the entire basal segment, are extremely rare. We report such a case of anomalous systemic arterial supply to the posterior basal segment of a normal lung in a patient with dextrocardia herein.

## CASE PRESENTATION

**Fig. 1:**
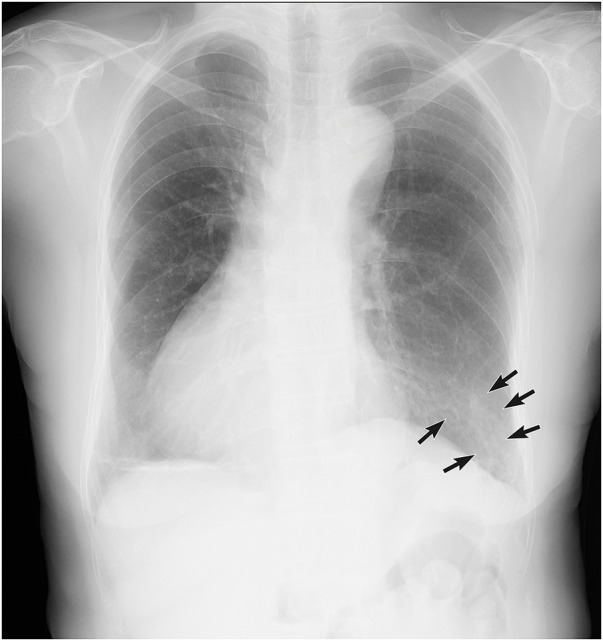
The chest X-ray shows a cord-like shadow in the left lower lung field, lateral region, continuous with a blood vessel (black arrows).

A 72-year-old female, a nonsmoker, with a history of colorectal cancer surgery 3 years prior and gastric cancer surgery 2 years prior, was on dipyridamole and aspirin for carotid arteriosclerosis. During a routine health checkup, a chest X-ray revealed a shadow on the periphery of the left lung, continuous with blood vessels, raising suspicion of an arteriovenous malformation (**Fig. 1**). She was then referred to our department. She exhibited no symptoms such as hemoptysis, cough, or exertional dyspnea at the time of consultation. The results of blood chemistry study showed no abnormalities.

**Fig. 2:**
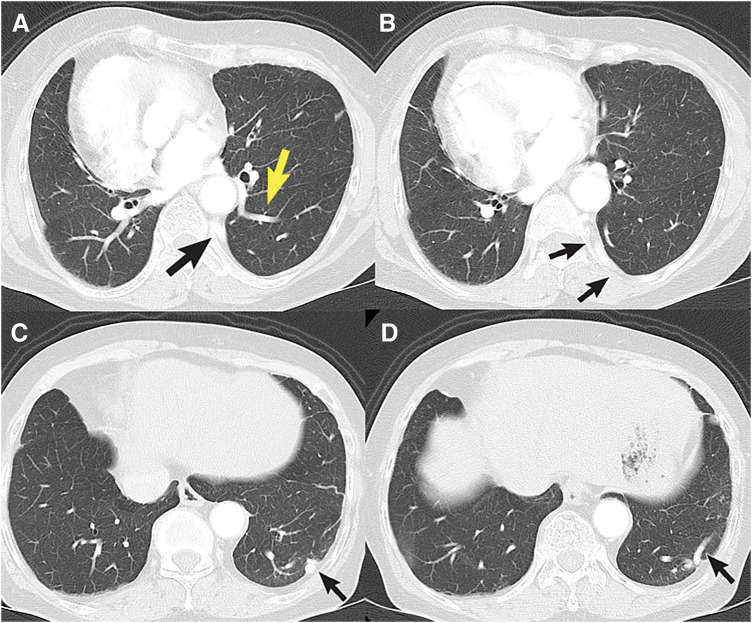
Chest CT (lung window). (**A**) Origin of the aberrant artery arising from the descending aorta (black arrow) and a dilated pulmonary vein branch (V10, yellow arrow). (**B**) Course of the aberrant artery running along the chest wall posterior to the parietal pleura (black arrows). (**C**) Entry point of the aberrant artery into the lung parenchyma (black arrow). (**D**) Intrinsic branches of the aberrant artery within the lung parenchyma (black arrow).

**Fig. 3:**
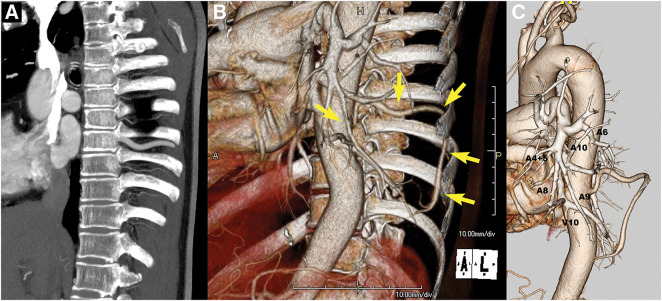
(**A**) Sagittal view of contrast-enhanced chest CT shows an abnormally thick 9th intercostal artery. (**B**) A 3D reconstruction image, overlapping surrounding structures such as the thoracic wall, shows the thick 9th intercostal artery branching from the descending aorta and an anomalous artery branching into the thoracic cavity (yellow arrows). (**C**) A 3D reconstruction focusing on the pulmonary and aortic systems shows almost normal branching of the left pulmonary artery to A6, A8, and A9. However, A10 demonstrates hypoplasia. While V10 is abnormally thick and well-developed, no vascular malformations, such as abnormal anastomoses or arteriovenous fistulas, are observed between the anomalous artery originating from the 9th intercostal artery and the pulmonary venous system.

Chest CT demonstrated an aberrant artery originating from the descending aorta (**Fig. 2A**), running along the chest wall posterior to the parietal pleura (**Fig. 2B**), and entering into the lung parenchyma (**Fig. 2C**), distributing to the S10 region (**Fig. 2D**). A dilated V10 branch was also visualized (**Fig. 2A**). A 3D reconstructed images of contrast-enhanced CT revealed dextrocardia and a thick anomalous artery branching from the aorta, running as the 9th intercostal artery (**Fig. 3A**), and supplying the S10 region of the left lung (**Fig. 3B**). Almost normal branching of the left pulmonary artery to A6, A8, and A9 was visualized; however, A10 demonstrated hypoplasia (**Fig. 3C**). Venous drainage was through the normal pulmonary vein system, especially via the V10 branch. While V10 was abnormally thick and well-developed, no vascular malformations, such as abnormal anastomoses or arteriovenous fistulas, were observed between the anomalous artery originating from the 9th intercostal artery and the pulmonary venous system.

**Fig. 4:**
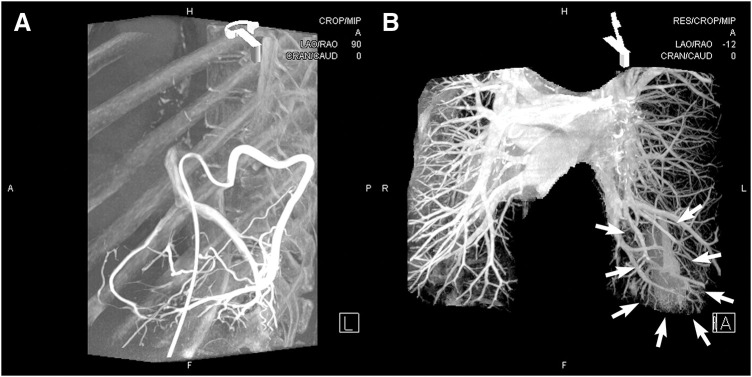
(**A**) Angiography reveals the left 9th intercostal artery, which develops an anomalous branch extending from the dorsal thoracic wall into the thoracic cavity, perfusing the left lung S10 region and draining through the V10 pulmonary vein branch into the left atrium. (**B**) In the venous phase, a highly stained area is observed, centered in the left lung S10 region (arrows).

Angiography revealed the left 9th intercostal artery, which developed an anomalous branch extending from the dorsal thoracic wall into the thoracic cavity, perfusing the left lung S10 region and draining through the V10 pulmonary vein branch into the left atrium (**Fig. 4A**). In the venous phase, a highly stained area was observed, centered in the S10 region (posterior basal segment), but no apparent sequestered lung tissue was identified (**Fig. 4B**).

Bronchoscopy demonstrated the absence of bronchial anomalies. On the basis of these findings, the case was diagnosed as anomalous systemic arterial supply to the posterior basal segment of a normal lung originating from the aorta, because the abnormal perfusion was limited primarily to the S10 region rather than the entire basal segment.

**Fig. 5:**
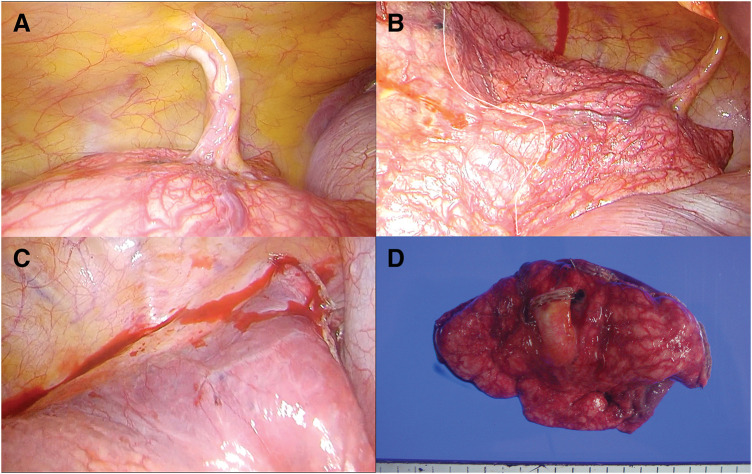
(**A**) Operative finding discloses an anomalous artery branching from the 9th intercostal artery and supplying the S10 region of the left lung. (**B**) The surface of the visceral pleura in the S10 region is covered with dilated vessels (right side from the white line). (**C**) Vascular dilation on the pleural surface of the residual lung quickly disappeared after resection of the anomalous artery and S10 region. (**D**) The macroscopic view of the specimen.

Due to the patient’s carotid arteriosclerosis and the risk of sudden death from hemoptysis, surgical intervention was deemed necessary. Surgery was performed via video-assisted thoracoscopic surgery through a small axillary thoracotomy. The left lung was unilobar. An anomalous artery branching from the 9th intercostal artery and supplying the S10 region of the left lung was identified (**Fig. 5A**). The surface of the visceral pleura in the S10 region was covered with dilated vessels (**Fig. 5B**). The anomalous artery was transected using the Ethicon Autosuture EndoGIA Ultra Tristapler camel reload 45 mm (Ethicon, New Jersey, USA). The visibly engorged and reddened areas on the visceral pleural surface corresponding to the S10 region were partially resected using 3 applications of the purple reload 60 mm stapler. Then, vascular dilation on the pleural surface of the residual lung quickly disappeared (**Fig. 5C**). The procedure was completed in 46 min, with a blood loss of 5 g. The macroscopic view of the specimen is shown in **Fig. 5D**.

**Fig. 6:**
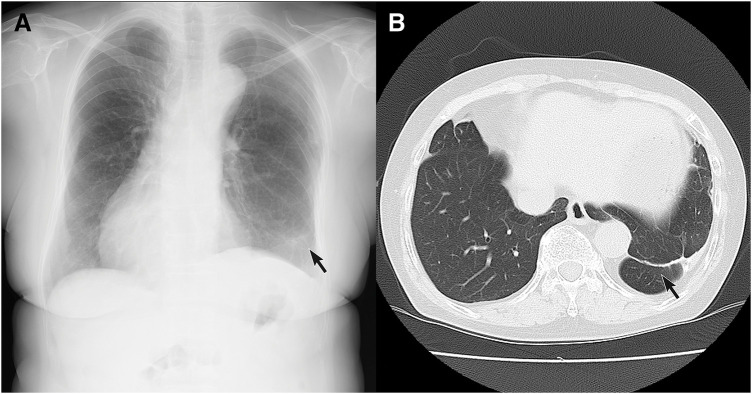
(**A**) Postoperative chest X-ray shows a staple line in the left S10 region (arrow). (**B**) Similarly, postoperative chest CT in lung window settings reveals a staple line in the left S10 region (arrow), with no evidence of abnormal pulmonary vascular engorgement.

Histopathological examination revealed normal bronchioles and alveolar structures, alongside irregular thickening and tortuosity of arterial and venous walls. Chest X-ray (**Fig. 6A**) and chest CT (**Fig. 6B**) taken 2 years after surgery showed a staple line in the right lower lobe but no abnormal vascular dilatation. The patient remains healthy and active, with no evidence of aneurysmal changes at the stapled end of the anomalous artery 7 years postoperatively.

## DISCUSSION

Dextrocardia refers to a condition in which the heart is positioned on the right side of the thorax. Based on the arrangement of other organs (situs), it is classified into 3 types: situs solitus (normal organ arrangement), situs inversus (complete organ mirror-image arrangement), and situs ambiguous (irregular or unclear organ arrangement). Isolated dextrocardia, with normal organ arrangement, occurs in situs solitus. Conversely, complete organ mirror-image reversal is termed situs inversus, and dextrocardia in such cases is referred to as mirror-image dextrocardia. Situs ambiguous involves heterotaxy, often associated with asplenia or polysplenia.^3)^

Isolated dextrocardia is associated with congenital heart defects, anomalies of the pulmonary venous system, and pulmonary hypoplasia. However, to our knowledge, this is the 1st report of a pulmonary posterior basal segmental artery originating from the aorta in isolated dextrocardia without abnormalities in the pulmonary venous system. This case provides significant insight into its embryological basis.

In cases of a pulmonary basal segmental artery originating from the aorta, shunting occurs due to blood flow supplied by a systemic artery and drained into the pulmonary vein. This results in volume overload and may lead to pulmonary hypertension. Over time, this can cause hemoptysis or heart failure, depending on the extent of the perfused region. While surgical intervention is considered standard for all cases, whether to operate on asymptomatic patients remains debated.^4)^

Most anomalous arteries arise from the descending aorta.^5)^ In this case, the 9th intercostal artery presented as the anomalous artery, forming an independent branch that perfused the lung after traversing the intrathoracic cavity. Unlike most reports where the anomalous artery enters the lung via the hilum or pulmonary ligament, this case involved transpleural entry of the artery into the lung, a finding reported only once before.^6)^ The perfusion region was also limited to the S10 region rather than the entire basal segment, which is unusual for pulmonary basal segmental artery anomalies.

Surgical resection for pulmonary basal segmental artery anomalies typically involves lobectomy.^7)^ The main purpose of surgical intervention in such cases is to prevent local chronic pulmonary hypertension or hemoptysis caused by aberrant systemic arterial inflow. Therefore, precise anatomical resection of the entire perfused area may not be mandatory, as long as the aberrant artery is securely ligated and the presumed area of abnormal perfusion is adequately resected. In the present case, rather than performing a formal anatomical segmentectomy, we opted for a wedge resection based on intraoperative assessment, including discoloration and vascular engorgement observed on the visceral pleural surface, to approximate the perfusion area. Therefore, only the visibly engorged and discolored areas on the lung surface were resected. Preserving normal lung tissue is desirable whenever feasible, making the primary surgical approach the disconnection of the anomalous artery and resection of the perfused lung tissue.

Although intraoperative use of indocyanine green has been reported for delineating the resection line,^8)^ it may not be necessary in most cases where surface vessels are visibly engorged and discolored. Regarding the treatment of anomalous arteries, stapling or ligation remains debated. Our prior experience with a 10-year follow-up of another pulmonary basal segmental artery anomaly case demonstrated no aneurysmal changes after stapling the artery near its aortic origin.^9)^ This is consistent with reports of successful arterial stapling in renal cancer cases involving the renal artery, suggesting that stapling is a safe and effective approach. Indeed, most reported cases of pulmonary basal segmental artery anomalies have utilized stapling for anomalous artery management.^5)^

## CONCLUSIONS

We experienced a pulmonary artery originating from the aorta with perfusion only in a part of the basal segment of the lung complicated by a right thoracic heart. When preoperative angiography showed only segmental stain, it is considered safe and sufficient to resect only to the extent of the surface vasodilatation grossly during surgery.

## DECLARATIONS

### Funding

No funding was involved in this case report.

### Authors’ contributions

AY and TI: Drafting of the manuscript and acquisition of the data.

KH, HI, MT, RI, TT, RM, and SH: Critical revision of the manuscript.

TI: Final approval of the manuscript.

All authors are in agreement regarding the content of the manuscript.

All authors read and approved the final manuscript.

### Availability of data and materials

The data that support the findings of this study are available from the corresponding author upon reasonable request.

### Ethics approval and consent to participate

This work does not require ethical considerations or approval.

### Consent for publication

Written informed consent was obtained from the patient for the publication of this case report.

### Competing interests

The authors declare that they have no competing interests.
